# Genetic Characterization of *Staphylococcus aureus* Isolated from Retail Meat in Riyadh, Saudi Arabia

**DOI:** 10.3389/fmicb.2016.00911

**Published:** 2016-06-09

**Authors:** Muhabat A. Raji, Ghada Garaween, Ralf Ehricht, Stefan Monecke, Atef M. Shibl, Abiola Senok

**Affiliations:** ^1^Department of Microbiology & Immunology, College of Medicine, Alfaisal UniversityRiyadh, Saudi Arabia; ^2^Alere Technologies GmbHJena, Germany; ^3^InfectoGnostics Research CampusJena, Germany; ^4^College of Pharmacy, King Saud UniversityRiyadh, Saudi Arabia; ^5^Infection and Immunity Department, King Faisal Specialist Hospital and Research CentreRiyadh, Saudi Arabia

**Keywords:** CC15-MRSA, camel meat, retail meat, MRSA prevalence, Saudi Arabia

## Abstract

Limited data exist from the Gulf Cooperation Council states on the prevalence and population dynamics of *Staphylococcus aureus* colonizing livestock or contaminating retail meat. This study was designed to determine the presence and genetic characteristics of *Staphylococcus aureus* isolated from raw retail meat sold in Riyadh, Saudi Arabia. Over a period of 9 months, different raw retail meat types were aseptically processed using the double broth enrichment technique, characteristic colonies from chromogenic and mannitol salt agar were further identified using conventional methods. Susceptibility to 9 antibiotics was determined using the disc diffusion technique. Interpretation of inhibition zone was done according to Clinical and Laboratory Standards Institute guidelines. Molecular characterization was carried out using the StaphyType DNA microarray technology. Twenty-five meat samples yielded *Staphylococcus aureus* isolates. Camel meat had the highest contamination rate with Methicillin resistant *Staphylococcus aureus* (MRSA) (20%) and Methicillin susceptible *Staphylococcus aureus* (28%), while poultry meat had the least contamination rate with MRSA (4%). The MRSA isolates were grouped into 4 clonal complexes (CCs) namely CC1-MRSA-IV/SCCfus (*n* = 2), CC15-MRSA-V/SCCfus (*n* = 4), CC80-MRSA-IV/PVL+ (*n* = 5), and CC88-MRSA-IV/PVL+ (*n* = 2). All CC15-MRSA-V/SCCfus isolates were obtained from camel meat. This is the first study to demonstrate the novel CC15-MRSA-V/SCCfus in retail camel meat. We recommend that surveillance studies should be incorporated in public health and food hygiene programs.

## Introduction

First identified in the hospitals, later in the community and livestock, the epidemiology of methicillin resistant *Staphylococcus aureus* (MRSA) is dynamic and continues to evolve. The zoonotic potential of these bacteria is now recognized (Petinaki and Spiliopoulou, 2012). Human acquisition of livestock associated methicillin resistant *Staphylococcus aureus* (LA-MRSA) could occur as a result of direct contact with livestock, contaminated environment or through contaminated retail meat (Graveland et al., 2011; Petinaki and Spiliopoulou, 2012; Greeson et al., 2013; Monaco et al., 2013; Visciano et al., 2014).

ST398, although notoriously associated with pigs, have also been described in veal calves, in cases of bovine mastitis and in poultry (including chickens and especially turkeys) (Fluit, 2012). In a 2011 study characterizing MRSA from food and food products in Germany, 28 out of 32 MRSA isolates were ST398 (Feßler et al., 2011). ST398 MRSA are associated with several distinct staphylococcal protein A (*spa*) types such as t011, t034, t108, t1451 (Köck et al., 2013). Apart from the heterogeneity in *spa* typing, staphylococcal cassette chromosome *mec* (SCC*mec*) typing also varies in identified strains, with most isolates harboring either SCC*mec* IV or V (Feßler et al., 2011; Graveland et al., 2011; Köck et al., 2013). However, some LA-MRSA isolates from China (Cui et al., 2009; Graveland et al., 2011) and Germany (Feßler et al., 2011) contained SCC*mec* III. Other LA-MRSA have been identified that belong to clonal complexes (CC) CC9, CC5, CC97, and CC30 (Feßler et al., 2011; Fluit, 2012). Distribution of clones also appear geographically diverse with ST398 being predominant in Europe, while most LA-MRSA from Asia are CC9 (Wagenaar et al., 2009; Petinaki and Spiliopoulou, 2012). The majority of LA-MRSA that have been studied so far lack the *pvl* and enterotoxin genes but demonstrate co-resistance to many non-beta-lactam antibiotics and heavy metals (Petinaki and Spiliopoulou, 2012). Recently, a homolog of the *mecA, mecC* (*mecA*_LGA251_), which is present on SCC*mec*XI, was identified in MRSA isolated from bulk milk (Garcia-Alvarez et al., 2011) and also in MRSA from human patients (Shore et al., 2011). It has been shown that isolates carrying the *mecC* gene can be exchanged between humans and ruminants (Petersen et al., 2012). These observations suggest that livestock could be a reservoir for novel clones of MRSA that may be transmitted to humans. *S. aureus* may also contaminate other food sources such as honey, Bezirtzoglou et al. isolated various multidrug resistant bacteria from honey in the Epirus region of Greece (Bezirtzoglou et al., 2016).

Limited data exist on MRSA contamination rate of retail meat, colonization of livestock and their molecular characteristics in the Gulf Cooperation Council (GCC) states including Saudi Arabia. This study was designed to determine the presence and genetic characteristics of *Staphylococcus aureus* (both methicillin susceptible and resistant) contaminating raw retail meat sold in Riyadh, Saudi Arabia.

## Methods

### Study design

The study was carried out from March-December 2014 in Riyadh Saudi Arabia. Randomly selected outlets of three major supermarket chains (RSC) and neighborhood meat shops (NMS) were included in the study. A total of 18 RSC outlets and 24 NMS were visited and these were equally distributed across the city. Targeted fresh retail meat type included camel, beef, lamb/mutton and chicken. Samples of each of these meat type were purchased weekly either from the RSC outlet or the NMS. The samples obtained from the RSC were bought in a pre-packaged form, those from the NMS were received into sterile dedicated containers at the point of purchase. Any of the targeted meat types available for sale on the day of visit to the RSC outlets or NMS were purchased. There was no duplication as samples were obtained once from each of the RSC outlets and NMS included in the study. All packages were transported in dedicated containers and were only opened for processing in the laboratory. All meat samples were received at the laboratory within 1 h of purchase.

### Sample design and sampling procedures

A total of one hundred meat samples were obtained and analyzed. Each raw meat sample was aseptically processed using the double broth enrichment technique modified from Jackson et al. (2013). Briefly, 250 g of meat sample was placed in a sterile bag and 100 ml of phosphate buffer saline (PBS) was added. The bag was shaken vigorously on a vortex mixer for 2 min to suspend bacteria from the meat surface. After vortexing of the PBS rinsate, a 1 ml aliquot was added to 9 ml of brain heart infusion broth (BHI) containing 6.5% salt (Saudi Prepared Media Limited [SPML] Riyadh, Saudi Arabia). The tubes were incubated for 24 h under aerobic conditions at 37°C; aliquots of 1 ml from turbid BHI tubes were added to 9 ml peptone water (PW) and incubated as described above. Sub-cultures were made from turbid PW tubes onto MRSA chromogenic agar (SPML) and mannitol salt agar (SPML) using sterile microbiological loops; thereafter the plates were incubated aerobically at 35°C for 24 h.

### Isolation of *S. aureus*

Pinkish colonies on MRSA chromogenic medium (presumptive MRSA) and yellowish colonies on mannitol salt agar (presumptive *S. aureus*), were further characterized using Gram stain, catalase, clumping factor/coagulase (latex; Staphauex Plus Remel, Dartford, England and tube; STAPH-ASE, bioMerieux, Marcy l'Etoile, France) tests and DNase agar (SPML). The susceptibility profile of the isolates to 9 antibiotics; Penicillin 10 units, Cefoxitin 30 μg, Gentamicin 10 μg, Erythromycin 15 μg, Clindamycin 2 μg, Ciprofloxacin 5 μg, Trimethoprim-Sulfamethoxazole 1.25/23.75 μg, Rifampin 5 μg and Linezolid 30 μg (Oxoid, Basingstoke, UK), was determined using the disc diffusion technique. Interpretation of inhibition zone was done according to Clinical and Laboratory Standards Institute guidelines (CLSI, 2013). The control strain (*S. aureus* ATCC 25923) was used in parallel in each run. Isolates were stored at −80°C for molecular characterization.

### Characterization of isolates

Molecular characterization was carried out using the StaphyType DNA microarray (Alere Technologies GmbH) technology as previously described (Monecke et al., 2008, 2011a, 2012). The DNA microarray targets over 170 genes including their allelic variants including the *mecA* (Monecke et al., 2008). Briefly, DNA samples from the organism grown on Colombia-blood agar were used as templates in a linear primer elongation using one primer target. Within this step, biotin-dUTP was incorporated into the resulting single stranded amplicons. These amplicons were hybridized to the microarray followed by washing and blocking steps. Then, horseradish-peroxidase-streptavidin conjugate was added. After further incubation and washing steps, hybridizations were visualized by adding a precipitating dye. Finally, an image of the microarray was taken and analyzed using the Arraymate reader and dedicated software (Alere Technologies GmbH). Normalized intensities of the spots were calculated considering gray values of the spots as well as of the local background. The presence or absence of target genes, assignment to CC as previously defined by MLST and eBURST (Based Upon Repeat Sequence Types) analysis of MLST was done (Monecke et al., 2008, 2011a, 2012).

## Results

A total of 100 meat types were obtained comprising of 29 poultry (chicken parts), 24 camel, 24 lamb/mutton and 23 beef. The distribution of meat type and source is shown in Table 1. Twenty-five samples (25%) yielded *S. aureus* isolates out of which 52% (n/N 13/25) were MRSA and the rest were methicillin susceptible *S. aureus* (MSSA; n/N 12/25). The overall contamination rate with MRSA was 13% (n/N 13/100). Camel meat had the highest contamination rate of MRSA (5/25, 20%) and MSSA (7/25, 28%), while poultry meat had the least contamination rate of MRSA (1/25, 4%). Not all meat types purchased on the same day and time from the same store yielded MRSA/MSSA. Table 2 gives the prevalence and distribution of MRSA and MSSA isolates among the different meat samples. None of the retail meat samples analyzed yielded a combination of both MSSA and MRSA isolates.

**Table 1:** **Distribution and source of the MRSA/MSSA isolates**.

**Clonal complex and strain assignment**	**Number**	**Distribution (n, Source)**
CC1-MRSA-IV/SCC*fus*	2	Beef (1, NMS), poultry (1, RSC)
CC15-MRSA-V/SCC*fus*	4	Camel (4, NMS)
CC80-MRSA-IV/PVL+	5	Camel (1,NMS), lamb (3: 2, RSC; 1, NMS), beef (1, NMS)
CC88-MRSA-IV/PVL+	2	Beef (1, NMS), lamb (1, NMS)
CC5-MSSA	2	Poultry (2: 1, NMS; 1, RSC)
CC15-MSSA	1	Camel (1, NMS)
CC88-MSSA/PVL+	1	Poultry (1, NMS)
CC101-MSSA	1	Camel (1, NMS)
CC121-MSSA/PVL+	1	Poultry (1, NMS)
CC361-MSSA	2	Camel (1, NMS), lamb (1, NMS)
ST1156-MSSA	4	Camel (4, NMS)
	25	21 NMS, 4 RSC

*Key: NMS, neighborhood meat shop; RSC, retail supermarket chain*.

**Table 2:** **Prevalence and distribution of MRSA/MSSA among the meat samples**.

**Meat type**	**Number of samples**	**No. (%) of samples positive**	
		**MSSA**	**MRSA**
Poultry	29	4 (16)	1 (4)
Camel	24	7 (28)	5 (20)
Lamb/mutton	24	1 (4)	4 (16)
Beef	23	0 (0)	3 (12)
	100	12 (48)	13 (52)

The MRSA isolates were grouped into 4 clonal complexes (CCs) namely CC1-MRSA-IV/SCC*fus* (*n* = 2), CC15-MRSA-V/SCC*fus* (*n* = 4), CC80-MRSA-IV/PVL+ (*n* = 5), and CC88-MRSA-IV/PVL+ (*n* = 2). In contrast there was a wider clonal diversity among the MSSA isolates which were grouped into 7CCs: CC5 (*n* = 2), CC15 (*n* = 1), CC88 (*n* = 1), CC101 (*n* = 1), CC121 (*n* = 1), CC361 (*n* = 2), and ST1156 (*n* = 4). All CC15-MRSA-V/SCC*fus* and ST1156 isolates were obtained from camel meat. All MRSA and MSSA isolates were susceptible to rifampin, linezolid and trimethoprim-sulfamethoxazole but there was variable susceptibility to the other antibiotics as shown in Table 3.

**Table 3:** **Number and percentage resistance to tested antibiotics**.

**Antibiotics**	**MSSA**	**MRSA**
	**n = 12 (%)**	**n = 13 (%)**
Penicillin	10 (83.3)	11 (84.6)
Cefoxitin	0 (0)	10 (76.9)
Ciprofloxacin	8 (66.7)	2 (15.4)
Erythromycin	5 (41.7)^*^	3 (23.1)^*^
Clindamycin	5 (41.7)^*^	3 (23.1)^*^^#^
Trimethoprim- sulfamethoxazole	0 (0)	0 (0)
Gentamicin	0 (0)	1 (7.7)
Linezolid	0 (0)	0 (0)
Rifampin	0 (0)	0 (0)

* Inducible resistance to clindamycin;

# *Intermediate resistance to clindamycin*.

### CC1 MRSA

Two isolates belonging to this clonal type were recovered. These isolates were both accessory gene regulator (*agr*)III, capsular antigen (*cap*)8, and enterotoxin (*ent*)H positive. Other immune evasion cluster genes associated with haemolysin-converting phages present in these isolates are presented in Table 4.

**Table 4:** **Distribution of selected virulence and resistance genes among the *Staphylococcus aureus* isolates**.

**Clonal type**	***n***	***agr***	***Cap***	**Virulence genes**	**Resistance genes**	**Others**
**MRSA CLONAL TYPES: 13 ISOLATES**						
CC1-MRSA-IV/SCC*fus*	2	III	8	hld, hla, hlb^*^, hlgA, clfA, clfB, lukD, lukF, lukS, lukE, lukX, lukY, entA, entH, entK^*^, entQ^*^	blaZ, blaI, blaR, ermC^*^, Q6GD50, mecA	*cna, isaB, sak, scn*
CC15-MRSA-V/SCC*fus*	4	II	8	*hld, hlgA, lukF, lukS, lukD, lukE, lukX, lukY, clfA, clfB*	blaZ, blaI, blaR, linA^*^, aacA-aphD, aadD, fosB Q6GD50, mecA	scn, chp, fib, fnB^*^, isaB^*^
CC80-MRSA-IV PVL^+^	5	III	8	*hld, hla, hlgA lukF, lukS, lukD, lukE, lukX, lukY, lukF-PV, lukS-PV, clfA, clfB*	*blaZ, blaI, blaR, tetK, aphA3, far 1, sat, mecA*	*sak, scn, etD, edinB, fib*
CC88-MRSA-IV PVL^+^	2	III	8	*hld, hlgA, hla, entA, lukK, lukQ, lukF, lukS, lukD, lukE, lukX, lukY, lukF-PV, lukS-PV, clfA, clfB*	*blaZ, blaI, blaR, ermC, mecA*	*sak, chp, scn, isaB*
**MSSA CLONAL TYPES: 12 ISOLATES**						
CC5-MSSA	2	II	5	*hld, hlgA, hla, entG, entI, entM, entO, entU, lukF, lukS*	*fosB*	*sak, scn*
CC15-MSSA	1	II	8	*hld, hla, lukD, lukE, lukX, lukY*	*blaZ, blaI, blaR, fosB*	*chp, scn*
CC88-MSSA PVL^+^	1	III	8	*hld, hlgA, hla, lukF, lukS, lukD, lukE, lukX, lukY, lukS-PV, lukF-PV, clfA, clfB, fib*	*blaZ, blaI, blaR, fosB*,	*sak, chp, scn*
CC101-MSSA	1	I	8	*hld, hlgA, hla, lukF, lukS, lukD, lukE, lukX, lukY, clfA, clfB, fib*	*fosB*	*sak, scn*
CC121-MSSA PVL^+^	1	IV	8	*hld, hla, hlgA, entB, entG, entI, entM, entN, entO, entU, lukF-PV, lukS-PV, l*uk*F, entS, entD, entE, entX, entY*	*blaZ, blaI, blaR, fosB, tetK*	*sak, scn*
CC361-MSSA	2	I	8	*hld, hla, entG, entI, entM, entN, entO, entU, lukF, lukS, lukD, lukE, lukX, lukY*	*blaZ, blaI, blaR, tetK, fosB*	*sak, scn*
ST1156-MSSA	4	I	8	*hld, hla, lukF, lukS, lukD, lukE, lukX, lukY, clfA, clfB*,	*blaZ, blaI, blaR, ermC, fosB*	*sak, scn, fib*

Key:

* *implies presence of gene is variable among the isolates, hld, haemolysin delta; hla, haemolysin alpha; hlb, haemolysin beta; hlgA, haemolysin gamma, component A, clfA, clumping factor A; clfB, clumping factor B; lukD, leukocidin D component; lukF, haemolysin gamma/leukocidin, component B; lukS, haemolysin gamma/leukocidin, component C; lukE, leukocidin E component; lukX, leukocidin/haemolysin toxin family protein; lukY, leukocidin/haemolysin toxin family protein; entA, B,G, H, I, K, M, N, O, Q, U, enterotoxin A, B, G, H, I, K, M, N, O, Q, U; lukS-PV, Panton Valentine leukocidin S component; lukF-PV, haemolysin gamma/leukocidin component B; fib, fibrinogen binding protein; blaZ, beta-lactamase; blaI, beta lactamase repressor; blaR, beta-lactamase regulatory protein; fosB, metallothiol transferase; ermC, erythromycin/clindamycin resistance; tetK, tetracycline-resistance; mecA, alternate penicillin binding protein 2, defining MRSA; aacA-aphD, bifunctional enzyme Aac/Aph, gentamicin resistance; aadD, aminoglycoside adenyltransferase, tobramycin resistance; Q6GD50, hypothetical protein associated with fusidic acid resistance; cna, collagen-binding adhesin; isaB, immunodominant antigen B; sak, staphylokinase; scn, Staphylococcal Complement inhibitor; chp, chemotaxis-inhibiting protein (CHIPS); etD, exfoliative toxin D; edinB, epidermal cell differentiation inhibitor B; linA, Lincosamide nucleotidyl transferase; agr, accessory gene regulator; n, number of isolates belonging to the specified clone*.

### CC15-MRSA/MSSA

Five isolates of this clonal type were isolated in the study; 4 MRSA and 1 MSSA. All isolates possed *agr*II. The four CC15-MRSA isolates carried SCC*mec*V and the SCC*fus* determinant which were not observed in previously reported human CC-15 MRSA isolates. Three of the CC15-MRSA isolates were phenotypically susceptible to cefoxitin but positive for *mecA*. There was variable penicillin resistance among these 3 CC15-MRSA isolates as only 1 isolate was resistant to penicillin. Furthermore, 2 CC15-MRSA-V/SCC*fus* carried the *linA* resistance determinant. All 4 isolates carried the *aadD* and *aacA-aphD* aminoglycoside resistance genes. The *ermC* gene was present in only 1 of the CC1-MRSA isolate. Variation was also observed in the distribution of other regulatory, virulence genes and antibiotic resistance determinants among the isolates as shown in Table 4.

### CC80-MRSA

These isolates were *pvl* positive and possessed *cap*8 and *agr*III determinants. Other genetic characteristics are presented in Table 4. There has been previous report of this strain from Saudi Arabia although it is referred to as the European community associated (CA)-MRSA clone.

### CC88-MRSA/MSSA

Three isolates made up of 2 MRSA and 1 MSSA were isolated. All isolates were *pvl* and *agr*III positive. Other characteristics are presented in Table 4.

### CC5-MSSA

The two CC5-MSSA isolates were recovered from poultry meat (bought from NMS), were susceptible to all tested antibiotics. *agr*II was present in these isolates. Other characteristics are shown in Table 4.

### CC101

Only one isolate belonging to this clonal type was isolated. The isolate possessed the *agr*I and *cap*8 determinants. Other genetic determinants are presented in Table 4.

### CC121-MSSA

One isolate belonging to this clonal type was characterized. It possessed the *pvl* agrIV determinants. Other characteristics of this isolate are shown in Table 4.

### CC361-MSSA

Two isolates were recovered and they both possessed the *agr*I and *cap*8 determinants. Other genetic characteristics are shown in Table 4.

### ST1156-MSSA

Four isolates belonging to this clonal types were recovered. All isolates had the *agr*I and *cap*8 genes. Other characteristics are shown in Table 4.

The ACME gene and *mecC* were not detected in any of the isolates. With regards to the immune evasion genes, *sak* (staphylokinase) was present in 50% of CC1-MRSA isolates while the *cna* (collagen binding adhesion) was present in CC1-MRSA and CC101-MSSA only. All CC15-MRSA isolates lacked enterotoxin, *sak* and *cna* genes but *scn* (staphylococcal complement inhibitor) and *chp* (chemotaxis-inhibiting protein) genes were present. The complete genetic profile of all isolates, based on the microarray assay, is provided in Supplementary Table 1.

## Discussion

Our study provides the first data evaluating the distribution and molecular characteristics of *S. aureus* including MRSA in retail meat in Saudi Arabia. The contamination rate of *S. aureus* in retail meat from this study is similar to the previous report from Riyadh by Greeson et al. (2013) (25 vs. 24.6%). However, in their study, Greeson et al. did not screen for MRSA among the isolated *S. aureus* strains recovered (Alzohairy 2011). Our findings also show that camel meat has the highest contamination rate with MRSA/MSSA of all the retail meat analyzed. A previous study from Saudi Arabia, evaluating nasal samples collected from farm animals revealed that camels had the highest prevalence of MRSA colonization (Greeson et al., 2013).

A report from the United Arab Emirates (UAE) analyzing milk and necropsy samples from camels did not identify any MRSA isolates but reported the presence of various toxin genes and resistance markers in the recovered MSSA isolates (Monecke et al., 2011b). The UAE study compared with our study may suggest different *S. aureus* population structures between the UAE and Saudi Arabia's camel herds. Moreover, postmortem contamination of camel meat with a human strain could be another scenario and, therefore, humans should also be screened for the presence of CC15-MRSA-V/SCC*fus*. Other MRSA lineages which we have identified, i.e., CC1-MRSA, CC80-MRSA, and CC88-MRSA, are well characterized human-associated clonal types that have been described in the literature (Ghebremedhin et al., 2009; Monecke et al., 2011a; Raji et al., 2013), suggesting retail meat contamination with human strains. ST398 MRSA, notoriously associated with pigs and poultry, especially turkeys, was not found in this study. This is not surprising since data from most countries demonstrate a close association between this strain and pigs or turkeys and neither pig nor turkey farming is practiced in Saudi Arabia. The low occurrence of MRSA in chicken meat is also noteworthy. Although this observation is quite different from what is obtained in other industrialized countries where there is commercial poultry farming, the reasons for our observation are not clear, since local commercial poultry farms exist in Saudi Arabia. Based on the molecular characterization from this study, the first report on the association between CC15-MRSA/SCC*fus* isolates and camel meat has emerged. In addition, a review of the literature also indicates that this is the first report on the association of this rare clone of MRSA with retail camel meat. In humans, CC15-MSSA is ubiquitously associated with carriage but CC15-MRSA remains rare having only been described sporadically. In a genotypic analysis of about 3000 isolates of MRSA, representing a pooled collection of human isolates, Monecke et al. (2011a) did not identify any CC15-MRSA (Monecke et al., 2011a). An Italian study described nosocomial CC15-MRSA, but these isolates carried SCC*mec* I and lacked the SCC*fus* determinant (Campanile et al., 2009). Furthermore, 2 CC15-MRSA SCC*mec* IV isolates (Japoni-Nejad et al., 2013; Abou Shady et al., 2015) have been reported from two studies evaluating MRSA colonization prevalence rates in humans. The *sak, scn*, and *chp* gene carrying phages are known to truncate haemolysin beta (*hlb*) more commonly in human *S. aureus* isolates, all the CC15-MRSA isolated in our study carried the *scn* and *chp* genes but lacked the *sak* gene (a typical feature of human CC15-MSSA isolates). Although CC15 MSSA is a successful lineage in humans and it can be found virtually everywhere, available literature on MRSA of this clonal lineage is few. This may suggest that this strain may not have acquired SCC*mec* elements I to IV on a significant scale (Monecke et al., 2011b). The presence of the SCC*mec*V/*fus* element, could be the first observation of an emerging strain.

All CC1-MRSA, CC80-MRSA, and CC88-MRSA isolates from our study possess the SCC*mec*IV, typical of community associated MRSA, which may in part explain the susceptibility to most of the tested non-beta lactam antibiotics.

Some authors have reported cefoxitin susceptibility in some genetically characterized MRSA isolates (Jackson et al., 2013; Kumar et al., 2013; Pu et al., 2014). These strains, referred to as oxacillin-susceptible *mecA* positive *S. aureus* (OS-MRSA), have been reported in diverse geographical regions (Pournaras et al., 2013), however, this is the first report of such isolates from retail meat in Saudi Arabia. The reasons for the emergence of OS-MRSA are not clear but plausible explanation include amino acid mutations in the FemXAB proteins involved in cell wall synthesis (Pu et al., 2014) or interference between different gene regulator systems (Sabat et al., 2015).

The small size of SCC*mec*V which is usually associated with community associated MRSA (CA-MRSA) lineages, may in part explain the limited antimicrobial resistance spectrum to non-beta-lactam antibiotics: resistance to gentamicin was observed in only one isolate and intermediate resistance to clindamycin in another.

The presence of known human clonal types on retail meat is suggestive of contamination, from human sources, along the production line probably due to inadequate meat hygiene. As not all meat types purchased on the same day and time from the same store yielded MRSA, it is likely that the contamination occurred before the point of sale. A study from Saudi Arabia showed that *S. aureus* was the second most common agent of food borne illness in an outbreak (Al-Goblan and Jahan, 2010). Although there are no surveillance data on clonal types colonizing livestock in the country, the presence of enterotoxin genes (*ent*A, H, K, Q) in some of the isolates we have identified in retail meat is noteworthy and indicative of the potential public health threat these isolates pose. Some limitations of our work include our inability to evaluate nasal samples from livestock, livestock farmers and butchers to establish a human-to-livestock/livestock-to-human transmission and lack of data on antibiotic use in camel and poultry farms. Nasal swab data from meat handlers would have enabled us to identify the possible points of contamination and better explain the probable transmission dynamics. Further work on this aspect is warranted. Furthermore, data on the use of antibiotics in camel and poultry farming need to be evaluated in future studies.

In conclusion, we have reported the isolation and characterization of *S. aureus* (MRSA and MSSA) clones contaminating retail meat in a region of Saudi Arabia. Our study also revealed a higher contamination rate of retail camel meat by a novel strain, CC15-MRSA/SCC*fus*. We recommend that surveillance and epidemiological studies aimed at understanding the changing epidemiology of MRSA isolates from livestock and meat products should be incorporated in public health, food hygiene and safety programs.

## Author contributions

AR and AS conceived the study. AR and GG collected the samples and did the preliminary phenotypic characterization of the isolates. SM and RE performed the DNA microarray. AR, AS, SM, AMS, RE analyzed the microarray data. AR and AS wrote the manuscript with critical appraisal and contribution from all authors. All authors read the final version of the manuscript and all gave their approval.

## Funding

This work was supported by Alfaisal University (grant IRG 3131801011427 to AR). The funding body did not in anyway, influence the study design, data collection and analysis, writing of the report and the choice of journal for article publication.

### Conflict of interest statement

RE and SM are employees of Alere Technologies GmbH Löbstedter Straße, Jena, Germany. The other authors declare that the research was conducted in the absence of any commercial or financial relationships that could be construed as a potential conflict of interest.
